# Hepatocyte growth factor pathway expression in breast cancer by race and subtype

**DOI:** 10.1186/s13058-021-01460-5

**Published:** 2021-08-03

**Authors:** Gieira S. Jones, Katherine A. Hoadley, Linnea T. Olsson, Alina M. Hamilton, Arjun Bhattacharya, Erin L. Kirk, Heather J. Tipaldos, Jodie M. Fleming, Michael I. Love, Hazel B. Nichols, Andrew F. Olshan, Melissa A. Troester

**Affiliations:** 1 Department of Epidemiology, Gillings School of Global Public Health, University of North Carolina-Chapel Hill, 253 Rosenau Hall, CB #7435, 135 Dauer Drive, Chapel Hill, NC USA; 2 Department of Genetics, University of North Carolina-Chapel Hill, Chapel Hill, NC USA; 3 Lineberger Comprehensive Cancer Center, University of North Carolina-Chapel Hill, Chapel Hill, NC USA; 4 Department of Pathology and Laboratory Medicine, University of North Carolina-Chapel Hill, Chapel Hill, NC USA; 5 Department of Biostatistics, University of North Carolina-Chapel Hill, Chapel Hill, NC USA; 6 Department of Biological and Biomedical Sciences, North Carolina Central University, Durham, NC USA

**Keywords:** Breast cancer, Hepatocyte growth factor

## Abstract

**Background:**

African American women have the highest risk of breast cancer mortality compared to other racial groups. Differences in tumor characteristics have been implicated as a possible cause; however, the tumor microenvironment may also contribute to this disparity in mortality. Hepatocyte growth factor (HGF) is a stroma-derived marker of the tumor microenvironment that may affect tumor progression differentially by race.

**Objective:**

To examine whether an HGF gene expression signature is differentially expressed by race and tumor characteristics.

**Methods:**

Invasive breast tumors from 1957 patients were assessed for a 38-gene RNA-based HGF gene expression signature. Participants were black (*n* = 1033) and non-black (*n* = 924) women from the population-based Carolina Breast Cancer Study (1993–2013). Generalized linear models were used to estimate the relative frequency differences (RFD) in HGF status by race, clinical, and demographic factors.

**Results:**

Thirty-two percent of tumors were positive for the HGF signature. Black women were more likely [42% vs. 21%; RFD = + 19.93% (95% CI 16.00, 23.87)] to have HGF-positive tumors compared to non-black women. Triple-negative patients had a higher frequency of HGF positivity [82% vs. 13% in non-triple-negative; RFD = + 65.85% (95% CI 61.71, 69.98)], and HGF positivity was a defining feature of basal-like subtype [92% vs. 8% in non-basal; RFD = + 81.84% (95% CI 78.84, 84.83)]. HGF positivity was associated with younger age, stage, higher grade, and high genomic risk of recurrence (ROR-PT) score.

**Conclusion:**

HGF expression is a defining feature of basal-like tumors, and its association with black race and young women suggests it may be a candidate pathway for understanding breast cancer disparities.

## Introduction

The tumor microenvironment can promote carcinogenesis by secretion of stroma-derived factors that are master regulators of cell division, growth, motility, and morphology [1]. Hepatocyte growth factor (HGF) is one of the major components secreted by the tumor stroma that drives tumorigenesis [1–5]. Takayama et al. conducted an in vivo study in transgenic mice and found that overexpression of HGF in mammary cells led to neoplasms [5]. In breast cancer cell lines, activation of the HGF pathway via binding of HGF to its receptor c-MET can lead to increased cell survival, proliferation, and resistance to cancer inhibitors [1]. In breast tumors, clinical studies have correlated activation of the HGF pathway (as defined by *c-MET* over-expression) with increased tumor size, high tumor grade, and distant metastasis [6]. In addition, a meta-analysis indicated that *c-MET* overexpression was associated with overall and disease-free survival in breast cancer patients [6]. However, in clinical trials that target the HGF-c-MET pathway, there is a lack of biomarkers for HGF expression that accurately identify patients prone to respond to targeted therapy [7].

The HGF pathway may also play a role in breast cancer disparities by race. *HGF* germline variations that differ by race have been found to modulate the expression of HGF in blood, normal tissue, and breast cancer tumor samples [8]. Ma et al. found that African American breast cancer patients had a higher frequency of truncating mutations (51%) in the promoter region of *HGF* compared to Caucasian breast cancer patients (15%), and these mutations were shown to result in increased expression of HGF in breast cancer tissue. The truncating mutations were also discovered in the germline (normal breast and normal blood tissue) and were found to be associated with case vs. non-case status [8]. We previously published an HGF gene expression signature that was associated with poorer survival in a small study [9], but our study population had insufficient racial diversity to evaluate the role of HGF gene expression by race.

Black women experience 40% higher mortality from breast cancer compared to white women [10]. It is important to identify biological pathways that may contribute to these disparities, and due to its role in cancer progression and differences in its expression, HGF is a plausible contributor. To evaluate the role of HGF pathway activation in breast cancer disparities, we assessed a 38-gene HGF gene expression signature in invasive breast cancer cases in a population-based resource, the Carolina Breast Cancer Study. We sought to understand associations between the HGF pathway and demographic characteristics, clinical features, and tumor subtypes within this racially diverse population.

## Methods

### Study population

The Carolina Breast Cancer study population has been described in previous publications [11, 12]. In brief, CBCS is a population-based study that utilized rapid case ascertainment to identify breast cancer cases from the North Carolina Cancer registry. Phases 1 and 2 of CBCS were conducted between 1993 and 2001 in 24 counties, and phase 3 subsequently expanded the study to a total of 44 counties in 2008–2013. Inclusion criteria included women who were North Carolina residents, ages 20–74 years old. Black and younger women (age < 50) were oversampled using randomized recruitment methods. Informed consent was obtained from each participant. This study was approved by the University of North Carolina at Chapel Hill Office of Human Ethics and Institutional Review Board. In total, there are 4806 invasive breast cancer cases who were enrolled in the Carolina Breast Cancer Study (phases 1–3). Within this population, 1188 participants were removed due to inadequate tissue for analysis. Quality control analysis removed 241 participants for low-quality RNA. Of the 3377 participants, 1957 were analyzed on the HGF gene expression assay. Participants that were not included in the study did differ on certain clinical variables including smaller tumor size lower grade and lower stage. However, the clinical and demographic features of the analysis set were similar to the distribution of the Carolina Breast Cancer Study as a whole, except tumors with a higher grade were more likely to be sampled. For the purposes of this analysis, 1957 invasive breast cancer cases with expression data for the HGF signature from all 3 CBCS phases (phase 1: *n* = 252, phase 2: *n* = 454, phase 3: *n* = 1251) were included.

### Demographic and clinical characteristics

Home interviews were conducted by a trained nurse, and all demographic and lifestyle information was self-reported, except body mass index (BMI), which was calculated from body measurements obtained by the nurse. Clinical tumor characteristics (estrogen receptor status, progesterone receptor status, HER2 receptor status, combined tumor grade and AJCC stage) were obtained from medical records, pathology reports, and immunohistochemical staining analysis at the University of North Carolina at Chapel Hill. Combined tumor grade was only available for CBCS phase 1 and phase 3 tumors and was assigned by a single pathologist to respective grading categories using the Nottingham breast cancer grading system [13]. Similar distributions for grade were observed within CBCS phase 1 and phase 3 (chi-square *p* value = 0.07). Phase 2 participants were excluded from analyses of the association of HGF with tumor grade.

### Gene expression data

Gene expression analysis for CBCS was described in prior publications [14]. Briefly, formalin-fixed paraffin-embedded (FFPE) tumor specimens were used (*n* = 2 (1 mm) cores; *n* = 2(10 μm) FFPE slides). RNA was isolated from FFPEs using the Qiagen FFPE RNeasy isolation kit (Germantown, MD) and counted using Nanostring nCounter technology (Seattle, Washington). A custom code set used to measure the genes used in the PAM50 predictor (to characterize RNA-based intrinsic breast cancer subtypes, namely luminal A, luminal B, HER2-enriched, basal-like and normal-like), risk of recurrence score (ROR-PT), and the HGF 38-gene signature [14, 15]. The ROR-PT score predicts the risk of distant recurrence incorporating information on subtype, proliferation score, and tumor size [15, 16]. After quantification of the RNA targets, NanoString gene expression values were normalized as previously described with remove unwanted variation (RUV), using the RUVg function from the RUVSeq Bioconductor package [17, 18]. We controlled for unwanted technical variation using the set of housekeeping genes that had expression above background in > 98% of samples and the highest correlation with expression of other housekeeping genes (Spearman coefficient ≥ 0.85). Six out of eleven housekeeping genes on our codeset met this criterion, namely *GUSB*, *ACTB*, *GAPDH*, *PGK1*, *RPLP0*, and *SF3A1.* Ultimately, we removed 2 dimensions of unwanted variation with RUVg (*k* = 2). Data was median centered across genes for heatmap visualization in R studio 3.5.3.

### HGF 38-gene signature

A 38-gene hepatocyte growth factor signature was derived from a 280-gene HGF signature previously described by our research group in Casbas-Hernandez et al. [9]. This 280-gene signature was mapped to three public gene expression datasets [NK1295 [19], UNC337 [20], and Naderi and colleagues [21]], and 109 unique genes were identified across all three datasets and used to classify tumors [9]. Using the shrunken centroid method [22], we identified 38 genes that could recapitulate the classification of samples based on the 109-gene set. The 38-gene HGF classifier includes the following genes: *TMEM45B*, *AKR7L*, *AQP5*, *C1QTNF3*, *C2ORF27A*, *C4ORF31*, *C9ORF98*, *CAPN13*, *CASKIN1*, *CMYA5*, *DTX3*, *EFHD1*, *F7*, *FMNL2*, *FUT8*, *GCNT2*, *HRC*, *INPP4B*, *ISLR2*, *KCNMA1*, *KCNN4*, *KIF3A*, *MAGI2*, *MARVELD2*, *NME5*, *PKIB*, *PRRG2*, *PRRT2*, *PVRL2*, *REEP6*, *RIMS4*, *SCUBE2*, *SHROOM3*, *SKAP1*, *SYBU*, *TFF3*, and *TMSB15B*.

To classify each sample as HGF-positive or HGF-negative, the 38 gene signature was applied using a weighted sum score, created by summing the magnitude of the normalized, log2 transformed values of the 38 genes within the consolidated HGF signature, and multiplying upregulated genes by 1 and downregulated genes by − 1 to preserve the directionality of each gene in the reference signature from our training cohort (Eq. 1).

1 $$\sum {W}_g\ast {Z}_g$$

- *W* = weight of gene (− 1 or 1 based off prior knowledge of upregulation or downregulation in HGF signature)
- *g* = gene in HGF expression signature
- *Z* = gene expression of *g* in known HGF signature

Within the original HGF signature [9], there were 6 genes upregulated (*FMNL2*, *KCNN4*, *AQP5*, *GCNT2*, *TMSB15B*, and *DOCK3*), and 32 were downregulated by HGF. The HGF weighted sum score was dichotomized using the mclust R package version 5.4.5, which determines cutpoints for classification based on Gaussian mixture analysis [23]. For this analysis, HGF positivity was defined as having the directional expression profile of tumors that are responsive to HGF protein treatment in breast cancer cells as assessed in Casbas-Hernandez et al. [9]. The modified HGF expression signature was concordant with the original signature trained on TCGA data (data not shown, 86% agreement, *p* value < 0.001).

### Statistical analysis

Demographic variables including age at diagnosis (< 40, 40–49, 50+ years old), race (black, non-black), parity and breastfeeding (nulliparous, parous and never breastfed, parous and breastfed), and family history of breast cancer (yes or no) were defined as categorical variables. Body mass index (BMI) was a continuous variable but was stratified by menopausal status and defined as a categorical variable (BMI: normal/underweight [BMI < 25], overweight [30 > BMI > 25], obese [BMI > 30]). Clinical characteristics and tumor subtypes were defined as follows, based on the clinical record: estrogen receptor status [positive (> 10% positivity), negative (0% positivity), borderline (1–10% positivity—was not included in this analysis; set to missing (*n* = 43))], progesterone receptor status [positive (> 10% positivity), negative (0% positivity), borderline (1–10% positivity—was not included in this analysis; set to missing (*n* = 100))], HER2 receptor status (positive or negative), hormone receptor tumor type (hormone receptor-positive/HER2 negative, triple-negative breast cancer, hormone receptor-negative/HER2 positive), triple-negative status (non-triple-negative breast cancer, triple-negative breast cancer), and clinical stage (AJCC: stage I, stage II, stage III/IV).

RNA-based variables were defined as follows: PAM50 intrinsic subtypes [luminal A, luminal B, HER2-enriched, basal-like, and normal-like, basal-like status (basal vs. non-basal), ROR-PT score (high, medium/low), and HGF (positive, negative). Normal-like samples were assumed to have insufficient tumor cellularity to produce a tumor call and therefore were removed from analysis (*n* = 67).

The HGF gene signature was assessed for associations with demographic and clinical data using generalized linear models to determine relative frequency difference estimates for univariate and multivariable models [24]. The generalized linear models used an identity link function with a binomial distribution to calculate the relative frequency differences. Multivariable models were adjusted for either age, race, or both. Covariates (age and race) were based on literature review and directed acyclic graph analysis. Race stratified analyses were defined as black vs. non-black. However, sensitivity analysis conducted between black women (*n* = 1033) vs. white women (*n* = 879) did not statistically differ from white vs. non-black associations with the HGF signature. To retain power to examine associations of the HGF signature with breast cancer features, all subsequent analysis combined white and “other” racial groups into the non-black category (“other race” *n* = 45). To address multiple hypothesis testing for associations of HGF with patient and clinical features, we used the Benjamini-Hochberg false discovery rate (FDR) method to test for multiple comparisons for all RFD models [25]. Statistical analysis was completed in both Stata 15 SE and R statistical environment version 3.5.3.

## Results

**Fig. 1:**
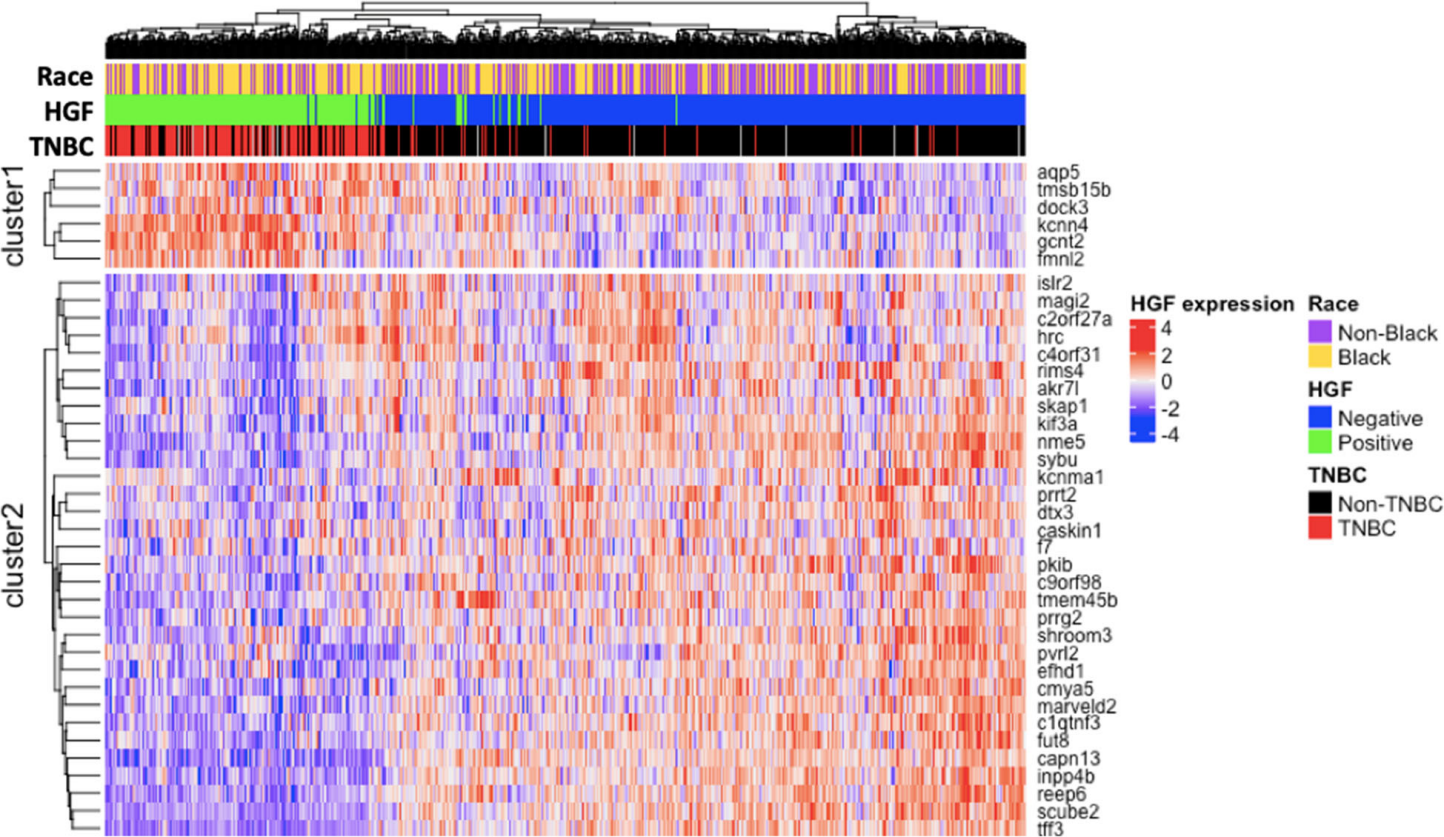
Heatmap of the 38-gene HGF gene signature in the Carolina Breast Cancer Study. Expression of genes in the HGF (hepatocyte growth factor) classifier clustered by gene and sample in CBCS, with indicators for race and triple- negative breast cancer (TNBC) subtype

In the Carolina Breast Cancer Study, 32% of participants were classified as HGF-positive by our 38-gene assay. Clustering the HGF signature genes across all of the CBCS patients, we found two main gene clusters that corresponded well with expression patterns from the original reference signature [9]. Specifically, HGF-positive tumors had few (*n* = 6) genes highly expressed, while most genes had a characteristic pattern of lower expression (Fig. 1). TNBC samples were enriched in the HGF-positive cluster.

To identify demographic characteristics associated with HGF-positive tumors, we evaluated relative frequency differences by race, age, and other variables. Table 1 shows that HGF positivity was more common among black women (compared to non-black women) [42% vs. 21%; RFD = + 19.93%, 95% CI (16.00, 23.87)]. Compared to women over 50, women under the age of 40 had the highest frequency of HGF-positive tumors [47% vs. 26%; RFD = + 20.33%, 95% CI (14.05, 26.61)], followed by women between the ages of 40–49 [[34% vs. 26%; RFD = + 8.51%, 95% CI (4.29, 15.98)]. Tumor grade III was strongly associated with HGF positivity [53% vs. 9%; RFD = + 41.11%, 95% CI (36.68, 45.55)] Higher breast cancer stage was also associated with increased prevalence of HGF positivity when compared to stage I [36% for stage II vs. 24%; RFD = + 6.16%, 95% CI (0.21, 12.11), and 37% for stage III/IV vs. 24%, RFD = + 7.89%, 95% CI (3.54, 12.22)]; however, after adjustment for multiple comparisons, associations with HGF and stage III/IV were no longer statistically significant (FDR *p* value = 0.068).

**Table 1:** Participant characteristics according to 38-gene HGF signature expression, Carolina Breast Cancer Study, 1993–2013

	HGF negative, *N* (%)	HGF positive, *N* (%)	Univariate RFD (95% CI)	Multivariable RFD (95% CI)
Total	1329 (68%)	628 (32%)		
Age^a^				
< 40 years old	1522 (53%)	100 (47%)	21.25% (14.81, 27.68)	20.33% (14.05, 26.61)
40–49 years old	498 (66%)	257 (34%)	8.25% (3.84, 12.66)	8.51% (4.29, 15.98)
≥ 50 years	679 (74%)	236 (26%)	Referent	Referent
Race^b^				
Black	603 (58%)	430 (42%)	20.20% (16.19, 24.20)	19.93% (16.00, 23.87)
Non-black	726 (79%)	198 (21%)	Referent	Referent
Grade^c^				
III	337 (47%)	384 (53%)	43.94% (39.76, 48.13)	41.11% (36.68, 45.55)
I/II	691 (91%)	71 (9%)	Referent	Referent
Stage^c^				
Stage III/IV	208 (64%)	115 (36)	11.56% (5.39, 17.71)	6.16% (0.21, 12.11)
Stage II	605 (63%)	349 (37%)	12.53% (8.06, 17.01)	7.89% (3.54, 12.22)
Stage I	499 (76%)	158 (24%)	Referent	Referent
Postmenopausal BMI^c^				
Obese	356 (73%)	132 (27%)	− 1.97 (− 9.10, 5.16),	− 6.23 (− 13.38, 0.92)
Overweight	209 (73%)	76 (27%)	− 2.35 (− 10.20, 5.50)	− 3.71 (− 11.24, 3.83)
Underweight/normal	159 (71%)	65 (29%)	Referent	Referent
Premenopausal BMI^c^				
Obese	225 (56%)	176 (44%)	16.07 (8.95, 23.20)	5.70% (− 1.68, 13.09)
Overweight	162 (65%)	86 (35%)	6.86 (− 1.02, 14.75)	2.62% (− 4.87, 10.11)
Underweight/normal	205 (72%)	79 (28%)	Referent	Referent
Reproductive history^c^				
Nulliparous	213 (71%)	85 (29%)	− 9.04% (− 15.07, − 3.02)	− 7.18% (− 12.91, − 1.46)
Parous, breastfed	551 (73%)	203 (27%)	− 10.64% (− 15.11, − 6.18)	− 6.77% (− 11.09, − 2.45)
Parous, never breastfed	565 (62%)	340 (38%)	Referent	Referent
Family history of breast cancer^c^				
Yes	235 (68%)	110 (32%)	− 0.18% (− 5.62, 5.26)	1.14.% (− 3.96, 6.25)
No	1053 (68%)	497 (32%)	Referent	Referent

^a^Relative frequency differences (RFDs) adjusted for race

^b^Adjusted for age

^c^Adjusted for race and age

Most breast cancer risk factors showed little association with HGF positivity. Body mass index and family history of breast cancer were not associated with HGF-positive tumors (Table 1). However, there was an inverse relationship between HGF-positive tumors and reproductive history factors. Specifically, nulliparous women and parous women with a history of breastfeeding had a lower frequency of HGF-positive tumors compared to parous women who have never breastfed.

Breast cancer subtypes are defined by tumor markers, clinically based on IHC or molecularly based on RNA expression, and both have been shown to have prognostic value. We considered both IHC-based and RNA-based subtypes in association with the HGF signature (Table 2). Among IHC-based subtypes, HGF positivity was strongly associated with triple-negative breast cancer, with 82% of these tumors having HGF positivity compared to 13% in non-TNBC [RFD = + 65.85, 95% CI (61.71, 69.98). When molecular subtypes were defined by PAM50, HGF positivity is a defining feature of basal-like subtypes. HGF positivity was present in 92% of basal-like, 32% in HER2-enriched, and only 6% or less in luminal subtypes (Table 2). HGF positivity was also significantly associated with high risk of recurrence scores (ROR-PT) in CBCS [71% vs. 19%; RFD = + 48.20%, 95% CI (43.42, 52.99)]. Given that HGF positivity was highly expressed in basal-like tumors, and basal-like status could be a mediator of the associations between HGF status and tumor aggressiveness, we performed a sensitivity analysis among non-basal tumors to see if the associations with HGF positivity and clinical characteristics remained. HGF positivity was still significantly associated with race, age, grade, and risk of recurrence scores within non-basal tumors, despite much lower prevalence of HGF positivity among these tumors (Table 3).

**Table 2:** Association of 38-gene HGF signature with breast cancer clinical and genomic subtypes and risk of recurrence (ROR) genomic score

	HGF negative, *N* (%)	HGF positive, *N* (%)	Multivariable RFD^a^ (95% CI)
Total (*N*)	1329 (68%)	628 (32%)	
**IHC-based**			
HR−/HER2+	62 (57%)	46 (43%)	29.42% (19.91, 38.93)
TNBC	86 (18%)	391 (82%)	69.04% (64.95, 73.14)
HR+/HER2−	1053 (89%)	125 (11%)	Referent
TNBC	86 (18%)	391 (82%)	65.85%. (61.71, 69.98)
Non-TNBC	1115 (87%)	171 (13%)	Referent
**RNA-based-PAM50**			
Luminal B	288 (94%)	19 (6%)	1.31% (− 1.51, 4.12)
HER2-enriched	122 (68%)	58 (32%)	27.29% (20.32, 34.27)
Basal-like	45 (8%)	493 (92%)	86.64% (83.73, 89.55)
Luminal A	820 (96%)	31 (4%)	Referent
Basal	45 (8%)	493 (92%)	81.84% (78.84, 84.83)
Non-basal	1230 (92%)	108 (8%)	Referent
High ROR-PT	145 (29%)	352 (71%)	48.20% (43.42, 52.99)
Low/medium ROR-PT	1133 (81%)	259 (19%)	Referent

^a^Relative frequency differences (RFDs) adjusted for age and race

*IHC*, immunohistochemistry; *ROR-PT*, risk of recurrence score incorporating subtype, proliferation, and tumor size

**Table 3:** Distribution of 38-gene HGF signature expression within non-basal-like tumors (*N* = 1338), Carolina Breast Cancer Study, 1993–2013

	Non-basal-like HGF-negative, *N* (%)	Non-basal-like HGF-positive, *N* (%)	Non-basal-like multivariable RFD (95% CI)
Total	1230	108	1338
Age^a^			
< 40 years old	141 (88%)	20 (13%)	5.40% (0.12, 10.67)
40–49 years old	458 (91%)	43 (9%)	2.06% (− 0.86, 4.98)
≥ 50 years old	631 (93%)	45 (7%)	Referent
Race^b^			
Black	561 (89%)	69 (11%)	5.35% (2.45, 8.26)
Non-black	669 (94%)	39 (6%)	Referent
Grade^c^			
III	296 (85%)	53 (15%)	10.37% (6.31, 14.42)
I/II	655 (96%)	25 (4%)	Referent
Stage^c^			
Stage III/IV	193 (86%)	31 (14%)	7.12% (2.25, 1.00)
Stage II	562 (92%)	52 (8%)	2.24% (− 0.60, 5.10)
Stage I	458 (95%)	24 (5%)	Referent
Reproductive history^c^			
Nulliparous	198 (93%)	14 (7%)	− 2.45% (− 6.32, 1.42)
Parous, breastfed	515 (90%)	39 (7%)	− 1.64% (− 4.7, 1.42)
Parous, never breastfed	517 (90%)	55 (10%)	Referent
Risk of recurrence score^c^			
High ROR-PT	128 (79%)	34 (21%)	13.56% (7.09, 20.02)
Low/medium ROR-PT	1063 (94%)	70 (6%)	Referent

^a^Relative frequency differences (RFDs) adjusted for race

^b^Adjusted for age

^c^Adjusted for race and age

*ROR-PT*, risk of recurrence score incorporating subtype, proliferation, and tumor size

## Discussion

This paper presents a novel biomarker of HGF positivity, a 38-gene signature developed through experimental methods and fine-tuned through application in breast tumor data. Using this signature, HGF positivity is associated with aggressive breast cancer subtypes and is strongly associated with basal-like subtype. In non-basal-like tumors, HGF positivity is less common; however, significant associations with many aggressive clinical features remained. Given that HGF positivity may ultimately be clinically targetable and is correlated with a number of poor prognosis clinical characteristics in both basal-like and non-basal-like tumors (including tumor stage, hormone receptor-negative markers, stage, tumor grade, and higher risk of recurrence scores), it is important to understand its distribution and contribution to outcomes. The distribution of HGF also highlights some long-standing breast cancer outcome disparities; HGF positivity is more prevalent among black participants and among women under the age of 50 (and especially those under 40). Taken together, this population-based study contributes important information on the distribution of HGF-positive tumors in breast cancer.

Our findings on the relationship between HGF and patient characteristics are consistent with previous literature on HGF, notably associations with black race, high stage, high grade, and younger age, but our findings extend the literature in several ways. First, previous studies focused mainly on germline genetic sequence and with relatively small patient numbers. Ma et al. observed that 51% of African American women had a higher frequency of mutations in the HGF promoter region when compared to 15% of Caucasian women. Our findings showed that not only does HGF positivity coincide with race at the somatic tumor gene expression level, but that HGF positivity is also associated with other features such as increased risk of recurrence (ROR) score and basal-like phenotype, providing a plausible link between HGF and racial disparities in breast cancer. In our study, we recognize that race is a social construct and understand this variable may encompass effects of environment, social inequities, and discrimination that are not captured in this analysis. However, we also note that self-reported race and ancestry are highly concordant in the CBCS population [26].

In our assessment of the association of age and HGF expression, women under the age of 50 had a higher frequency of HGF-positive tumors. Ma et al. also observed a statistically significant association with age, where younger breast cancer patients were more likely to have the HGF promoter mutation [8]. However, a recent meta-analysis of c-MET expression by Zhao et al. found no association between age and HGF expression [6]. The meta-analysis combined studies that used different methods to detect c-MET expression including protein(75% of studies) and RNA-based techniques(25% of studies) [6, 27], which could have contributed to between-study differences. Our analysis was concordant with a number of clinical studies [28–32], showing that higher stage and tumor grade were associated with HGF-positive tumors.

The HGF/c-MET axis is an attractive pathway in breast cancer research because it is targetable with existing therapeutics [1, 7, 30, 33]. Moreover, HGF positivity appears to be a feature of triple-negative/basal subtype [9, 31, 34–37], which does not currently have any targeted therapies [38]. However, the lack of an established HGF biomarker has been problematic. There are multiple biomarkers representing the HGF pathway in the literature including c-MET RNA expression, *c-MET*-exon skipping, c-MET protein expression, *c-MET* amplification, c-MET receptor, and/or HGF protein expression; however, they have not been validated and efficacy has only been proven in the *C-MET* exon skipping marker in clinical trials, where the demonstrated predictive benefit was minimal [7, 39]. Here we demonstrate a multi-gene HGF signature that can retain complex biological information on the pathway. This pathway could be targetable in both Basal-like and non-basal tumors. Although HGF-positive tumors were highly prevalent among triple-negative breast cancer; it was a defining feature among Basal-like tumors. Basal-like and triple-negative breast cancer subtypes are often used interchangeably, but there is heterogeneity in gene expression within triple-negative breast cancers [40, 41]. The associations with clinical characteristics and HGF positivity that we observed may have partially been mediated by the aggressive phenotype of the basal-like subtype.

HGF positivity also occurs in non-basal-like tumors. HGF positivity was present among 8% of non-basal-like tumors and was associated with more aggressive features, suggesting the pathway may also affect some of these tumors. Rahgav et al. examined the relationship between c-MET expression, as measured by reverse protein phase array, and breast cancer recurrence among 257 invasive breast cancers [42]. The study found that total c-MET levels in hormone receptor-positive and phosphorylated c-MET levels in HER2 subtypes were associated with recurrence [42]. This suggests that our findings that HGF positivity is associated with aggressive tumor phenotypes may have consequences for recurrence. Others have suggested that HGF c/MET expression may also influence prognosis specifically in HER2 overexpressing tumors via resistance to HER2-targeted therapies [43, 44]. These associations between HGF positivity and outcome should be assessed in future studies using the 38-gene assay developed here.

A strength of this analysis was the use of a novel 38-gene biomarker and a pathway-based approach, rather than classifying tumors based on a single gene. The signature was developed to be concordant with a larger signature in The Cancer Genome Atlas Project. Furthermore, the Nanostring technology has increased sensitivity and reproducibility when compared to traditional methods such as qPCR [45], particularly when using FFPE specimens. Another strength includes the large, racially diverse population-based study design. The large sample size lent itself to statistical power for the current analysis.

Some limitations also affect this work. While we describe the distribution of a novel signature for HGF, we lacked data to assess whether this signature predicted response to HGF therapy. We also do not have data on specific HGF-pathway proteins, impairing our ability to directly compare RNA vs. protein-based biomarkers. While we assayed RNA and did not specifically evaluate whether protein levels of HGF were concordant with RNA in this population, our previous research suggests that RNA-based findings were concordant with protein-based findings [9]. Therefore, the concordance of our findings with patterns in previous literature mitigates this concern somewhat. We were also unable to fully disentangle the role of basal-like subtype in driving HGF associations with tumor aggressiveness. The proportion of HGF positivity was so high among basal-likes and relatively uncommon among non-basal-likes, leaving these assessments somewhat underpowered, though even in these small strata, the associations with tumor aggressiveness appear consistent.

Currently, one of the leading challenges with targeting c-MET in clinical trials is the lack of selection of appropriate patient populations for targeted therapy [46]. There is a need for biomarkers to improve efficacy to target the c-MET/HGF signaling pathway, especially within breast cancer. Further validation of this novel biomarker could influence the use of the gene signature in identification for high-risk populations or for targeted treatment options.

## Conclusion

This study observed that the novel HGF gene expression signature was a defining feature in basal-like breast cancer tumors. This signature was also found to be more prevalent in women under 50 and black women, populations most severely affected by breast cancer outcome disparities. The prevalence of this signature among populations adversely affected by breast cancer suggests this pathway may be a candidate for targetable molecular therapy that influences breast cancer disparities.

## Data Availability

The datasets generated during the current study are available from the corresponding author upon reasonable request. The code in this study is available from the corresponding author upon reasonable request.

## Abbreviations

HGF: Hepatocyte growth factor
TNBC: Triple-negative breast cancer
RFD: Relative frequency differences
BMI: Body mass index
FFPE: Formalin-fixed paraffin-embedded
RUV: Remove unwanted variation

## References

[1] Parikh RA, Wang P, Beumer JH, Chu E, Appleman LJ. The potential roles of hepatocyte growth factor (HGF)-MET pathway inhibitors in cancer treatment. OncoTargets and Therapy. 2014. 10.2147/OTT.S40241 PMC4061161 24959084

[2] Owusu BY, Galemmo R, Janetka J, Klampfer L. Hepatocyte growth factor, a key tumor-promoting factor in the tumor microenvironment. Cancers. 2017;9(12). 10.3390/cancers9040035. 10.3390/cancers9040035 PMC5406710 28420162

[3] Haslam SZ, Woodward TL (2003). Host microenvironment in breast cancer development: epithelial-cell–stromal-cell interactions and steroid hormone action in normal and cancerous mammary gland. Breast Cancer Res.

[4] Eterno V, Zambelli A, Pavesi L, Villani L, Zanini V, Petrolo G, Manera S, Tuscano A, Amato A (2014). Adipose-derived mesenchymal stem cells (ASCs) may favour breast cancer recurrence via HGF/c-Met signaling. Oncotarget..

[5] Takayama H, Larochelle WJ, Sharp R, Otsuka T, Kriebel P, Anver M (1997). Diverse tumorigenesis associated with aberrant development in mice overexpressing hepatocyte growth factor/scatter factor. Proc Natl Acad Sci U S A.

[6] Zhao X, Qu J, Hui Y, Zhang H, Sun Y, Liu X, et al. Clinicopathological and prognostic significance of c-Met overexpression in breast cancer. Oncotarget. 2017. 10.18632/oncotarget.18142 PMC5593599 28915628

[7] Oliveres H, Pineda E, Maurel J (2020). MET inhibitors in cancer: pitfalls and challenges. Expert Opin Investig Drugs.

[8] Ma J, DeFrances MC, Zou C, Johnson C, Ferrell R, Zarnegar R (2009). Somatic mutation and functional polymorphism of a novel regulatory element in the HGF gene promoter causes its aberrant expression in human breast cancer. J Clin Invest.

[9] Casbas-Hernandez P, Troester MA, Perez ER, Sandhu R, Kirk E, D’arcy M (2012). Role of HGF in epithelial-stromal cell interactions during progression from benign breast disease to ductal carcinoma in situ. Cancer Res.

[10] American Cancer Society. Breast Cancer Facts & Figures 2019-2020. Am Cancer Soc. 2019.

[11] Newman B, Moorman PG, Millikan R, Qaqish BF, Geradts J, Aldrich TE, Liu ET (1995). The Carolina Breast Cancer Study: integrating population-based epidemiology and molecular biology. Breast Cancer Res Treat.

[12] Emerson MA, Golightly YM, Tan X, Aiello AE, Reeder-Hayes KE, Olshan AF, et al. Integrating access to care and tumor patterns by race and age in the Carolina Breast Cancer Study, 2008–2013. Cancer Causes Control. 2020. 10.1007/s10552-019-01265-0 PMC7188189 31950321

[13] Elston CW, Ellis IO (1991). Pathological prognostic factors in breast cancer. I. The value of histological grade in breast cancer: experience from a large study with long-term follow-up. C. W. Elston & I. O. Ellis. Histopathology.

[14] Troester MA, Sun X, Allott EH, Geradts J, Cohen SM, Tse CK, et al. Racial differences in PAM50 subtypes in the Carolina Breast Cancer Study. J Natl Cancer Inst. 2017;110(2). 10.1093/jnci/djx135 PMC6059138 28859290

[15] Parker JS, Mullins M, MCU C, Leung S, Voduc D, Vickery T (2009). Supervised risk predictor of breast cancer based on intrinsic subtypes. J Clin Oncol.

[16] Troester MA, Sun X, Allott EH, Geradts J, Cohen SM, Tse C-K (2018). Racial differences in PAM50 subtypes in the Carolina Breast Cancer Study. JNCI J Natl Cancer Inst.

[17] Bhattacharya A, Hamilton AM, Furberg H, Pietzak E, Purdue MP, Troester MA, et al. An approach for normalization and quality control for NanoString RNA expression data. bioRxiv. 2020; 10.1093/bib/bbaa163 PMC8138885 32789507

[18] Risso D, Ngai J, Speed TP, Dudoit S (2014). Normalization of RNA-seq data using factor analysis of control genes or samples. Nat Biotechnol.

[19] Van De Vijver MJ, He YD, van’t Veer LJ, Dai H, Hart AAM, Voskuil DW, et al. A gene-expression signature as a predictor of survival in breast cancer. N Engl J Med. 2002. 10.1056/NEJMoa021967 12490681

[20] Prat A, Parker JS, Karginova O, Fan C, Livasy C, Herschkowitz JI, et al. Phenotypic and molecular characterization of the claudin-low intrinsic subtype of breast cancer. Breast Cancer Res. 2010;12(5). 10.1186/bcr2635 PMC3096954 20813035

[21] Naderi A, Teschendorff AE, Barbosa-Morais NL, Pinder SE, Green AR, Powe DG, Robertson JFR, Aparicio S, Ellis IO, Brenton JD, Caldas C (2007). A gene-expression signature to predict survival in breast cancer across independent data sets. Oncogene..

[22] Tibshirani R, Hastie T, Narasimhan B, Chu G (2002). Diagnosis of multiple cancer types by shrunken centroids of gene expression. Proc Natl Acad Sci.

[23] Scrucca L, Fop M, Murphy TB, Raftery AE. Mclust 5: Clustering, classification and density estimation using Gaussian finite mixture models. R J. 2016;8(1). 10.32614/RJ-2016-021. PMC5096736 27818791

[24] Wacholder S (1986). Binomial regression in glim: estimating risk ratios and risk differences. Am J Epidemiol.

[25] Benjamini Y, Hochberg Y. Controlling the false discovery rate: a practical and powerful approach to multiple testing. J R Stat Soc Ser B. 1995.

[26] Bhattacharya A, García-Closas M, Olshan AF, Perou CM, Troester MA, Love MI (2020). A framework for transcriptome-wide association studies in breast cancer in diverse study populations. Genome Biol.

[27] Partridge AH, Hughes ME, Warner ET, Ottesen RA, Wong YN, Edge SB (2016). Subtype-dependent relationship between young age at diagnosis and breast cancer survival. J Clin Oncol.

[28] Sheen-Chen SM, Liu YW, Eng HL, Chou FF (2005). Serum levels of hepatocyte growth factor in patients with breast cancer. Cancer Epidemiol Biomark Prev.

[29] Yang H, Zhang C, Cui S. Expression of hepatocyte growth factor in breast cancer and its effect on prognosis and sensitivity to chemotherapy. Mol Med Rep. 2015. 10.3892/mmr.2014.2808 25351134

[30] Ho-Yen CM, Jones JL, Kermorgant S (2015). The clinical and functional significance of c-Met in breast cancer: a review. Breast Cancer Res.

[31] Ho-Yen CM, Green AR, Rakha EA, Brentnall AR, Ellis IO, Kermorgant S, et al. C-Met in invasive breast cancer: is there a relationship with the basal-like subtype? Cancer. 2014. 10.1002/cncr.28386 24150964

[32] Shin S, Ogawa M, Yamashita SI, Nomura K, Kuramoto M, Saishoji T. Immunoreactive hepatocyte growth factor is a strong and independent predictor of recurrence and survival in human breast cancer. Cancer Res. 1994. 8137271

[33] Comoglio PM, Giordano S, Trusolino L (2008). Drug development of MET inhibitors: targeting oncogene addiction and expedience. Nat Rev Drug Discov.

[34] Kim YJ, Choi JS, Seo J, Song JY, Eun Lee S, Kwon MJ, et al. MET is a potential target for use in combination therapy with EGFR inhibition in triple-negative/basal-like breast cancer. Int J Cancer. 2014. 10.1002/ijc.28566 24615768

[35] Graveel CR, DeGroot JD, Su Y, Koeman J, Dykema K, Leung S (2009). Met induces diverse mammary carcinomas in mice and is associated with human basal breast cancer. Proc Natl Acad Sci.

[36] Charafe-Jauffret E, Ginestier C, Monville F, Finetti P, Adélaïde J, Cervera N (2006). Gene expression profiling of breast cell lines identifies potential new basal markers. Oncogene.

[37] Breunig C, Erdem N, Bott A, Greiwe JF, Reinz E, Bernhardt S, Giacomelli C, Wachter A, Kanthelhardt EJ, Beißbarth T, Vetter M, Wiemann S (2018). TGFβ1 regulates HGF-induced cell migration and hepatocyte growth factor receptor MET expression via C-ets-1 and miR-128-3p in basal-like breast cancer. Mol Oncol.

[38] McCann KE, Hurvitz SA, McAndrew N (2019). Advances in targeted therapies for triple-negative breast cancer. Drugs.

[39] Koeppen H, Rost S, Yauch RL (2014). Developing biomarkers to predict benefit from HGF/MET pathway inhibitors. J Pathol.

[40] Lehmann BD, Bauer JA, Chen X, Sanders ME, Chakravarthy AB, Shyr Y (2011). Identification of human triple-negative breast cancer subtypes and preclinical models for selection of targeted therapies. J Clin Invest.

[41] Ring BZ, Hout DR, Morris SW, Lawrence K, Schweitzer BL, Bailey DB, et al. Generation of an algorithm based on minimal gene sets to clinically subtype triple negative breast cancer patients. BMC Cancer. 2016. 10.1186/s12885-016-2198-0 PMC4763445 26908167

[42] Raghav KP, Wang W, Liu S, Chavez-MacGregor M, Meng X, Hortobagyi GN, Mills GB, Meric-Bernstam F, Blumenschein GR, Gonzalez-Angulo AM (2012). cMET and phospho-cMET protein levels in breast cancers and survival outcomes. Clin Cancer Res.

[43] Minuti G, Cappuzzo F, Duchnowska R, Jassem J, Fabi A, Obrien T (2012). Increased MET and HGF gene copy numbers are associated with trastuzumab failure in HER2-positive metastatic breast cancer. Br J Cancer.

[44] Paulson AK, Linklater ES, Berghuis BD, App CA, Oostendorp LD, Paulson JE, Pettinga JE, Melnik MK, Vande Woude GF, Graveel CR (2013). MET and ERBB2 are coexpressed in ERBB2+ breast cancer and contribute to innate resistance. Mol Cancer Res.

[45] Tsang H-F, Xue VW, Koh S-P, Chiu Y-M, Ng LP-W, Wong S-CC. NanoString, a novel digital color-coded barcode technology: current and future applications in molecular diagnostics. Expert Rev Mol Diagn. 2017;17(1). 10.1080/14737159.2017.1268533 27917695

[46] Huang X, Li E, Shen H, Wang X, Tang T, Zhang X, et al. Targeting the HGF/MET axis in cancer therapy: challenges in resistance and opportunities for improvement. Frontiers in Cell and Developmental Biology. 2020;8:152. 10.3389/fcell.2020.00152 PMC7218174 32435640

